# Synthesis, Structure and Biological Evaluations of Zn(II) Pincer Complexes Based on S-Triazine Type Chelator

**DOI:** 10.3390/molecules27113625

**Published:** 2022-06-05

**Authors:** Heba M. Refaat, Atallh A. M. Alotaibi, Necmi Dege, Ayman El-Faham, Saied M. Soliman

**Affiliations:** 1 Department of Chemistry, Faculty of Science, Alexandria University, P.O. Box 426, Ibrahimia, Alexandria 21321, Egypt; atallh1415@gmail.com; 2 Department of Physics, Faculty of Arts and Sciences, Ondokuz Mayıs University, Samsun 55139, Turkey; necmid@omu.edu.tr

**Keywords:** Zn(II), pincer, *s*-triazine based ligand, Hirshfeld, anticancer, antimicrobial

## Abstract

2,4-*bis* (3,5-dimethyl-1*H*-pyrazol-1-yl)-6-methoxy-1,3,5-triazine (**BPMT**) pincer ligand was used to synthesize the new [Zn(BPMT)(NCS)_2_] (**1**) and [Zn(BPMT)(Br)_2_] (**2**) complexes by a reaction with Zn(NO_3_)_2_·6H_2_O in the presence of either KSCN or KBr, respectively. The structure of complex **1** has been exclusively confirmed using single crystal X-ray diffraction. In this neutral heteroleptic complex, the **BPMT** is a pincer chelate coordinating the Zn(II) ion via three interactions with the two pyrazole moieties and the *s*-triazine core. Hence, **BPMT** is a tridentate *NNN*-chelate. The coordination environment of Zn(II) is completed by two strong interactions with two terminal SCN^−^ ions via the *N*-atom. Hence, the Zn(II) is penta-coordinated with a distorted square pyramidal coordination geometry. Hirshfeld analysis indicated the predominance of H…H, H…C and N…H intermolecular interactions. Additionally, the S…H, S…C and S…N contacts are the most significant. The free ligand has no or weak antimicrobial, antioxidant and anticancer activities while the studied Zn(II) complexes showed interesting biological activity. Complex **1** has excellent antibacterial activity against *B. subtilis* (2.4 μg/mL) and *P. vulgaris* (4.8 μg/mL) compared to *Gentamycin* (4.8 μg/mL). Additionally, complex **1** (78.09 ± 4.23 µg/mL) has better antioxidant activity than **2** (365.60 ± 20.89 µg/mL). In addition, complex **1** (43.86 ± 3.12 µg/mL) and **2** (30.23 ± 1.26 µg/mL) have 8 and 12 times the anticancer activity of the free **BPMT** ligand (372.79 ± 13.64 µg/mL).

## 1. Introduction

**Figure 1:**
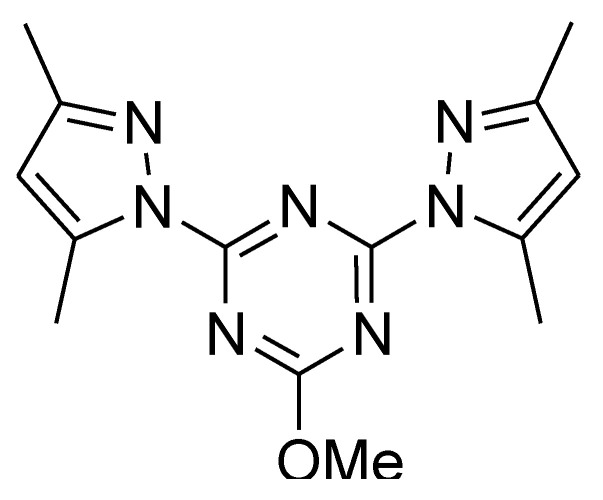
Structure of the ligand [11,12].

Triazines are known lead compounds which have a diverse range of pharmaceutical applications [1]. These heterocyclic compounds have interesting biological activities as antimicrobial [2], anticancer [3,4], anti-HIV [5,6], and anti-inflammatory activities [1]. Among the different types of triazines, 1,3,5-triazine (*s*-triazine) is a highly symmetrical molecule. In this regard, the self-assembly of such highly symmetrical ligands with metal salts is a simple way to build interesting supramolecular architectures for different applications, such as gas storage, catalysis, magnetism, nonlinear optics, and luminescence [7,8,9,10]. *s*-Triazine pincer ligands are an interesting class of polydentate ligands as they form stable complexes with different metal ions [11]. The *bis*-pyrazolyl-*s*-triazine ligand shown in Figure 1 attracted our attention due to its simple route of preparation and its interesting coordination chemistry with different metal ions [12,13,14,15].

Metal ions have an essential role in biological systems due to their interactions with many biomolecules, such as enzymes, serum albumin and amino acids [16]. For example, Zn(II) complexes have many applications as antidiabetic insulin mimetics [17], antimicrobial [18], and anticancer agents [19]. Additionally, these complexes attracted renewed attention due to their applications as tumor photosensitizers [20], radioprotective agents [21], and antidandruff agents [22]. Recently, the coordination chemistry of the pincer ligand, 2,4-*bis* (3,5-dimethyl-1*H*-pyrazol-1-yl)-6-methoxy-1,3,5-triazine (**MBPT**; Figure 1), was extensively studied by our research group [11,12,13,14,15,23,24]. Several low dimensional and polymeric complexes with different metal ions (Zn(II), Co(II), Cu(II)) and counter ions (Cl^−^, NO_3_^−^ and ClO_4_^−^) were synthesized and their biological activity was assessed as antimicrobial agents. The results revealed promising antimicrobial activities of these complexes against different microbes. In continuation to this work, two new Zn(II) complexes of **MBPT** ligand with different anions (SCN^−^ and Br^−^) were synthesized and characterized. In addition, their biological activities (antimicrobial, anticancer and antioxidant) were examined.

## 2. Results and Discussion

### 2.1. Synthesis and Characterizations

The reaction of an aqueous solution of Zn(NO_3_)_2_.6H_2_O with an ethanolic solution of the pincer ligand **BPMT** in presence of either KSCN or KBr afforded two new Zn(II) complexes. Due to the relatively weak coordinating power of the nitrate ion compared with the SCN^−^ and Br^−^ anions, the corresponding [Zn(BPMT)(NCS)_2_] (**1**) and [Zn(BPMT)(Br)_2_] (**2**) complexes were obtained, respectively. The structures of the two complexes were characterized using FTIR spectra and elemental analysis. The FTIR spectra of both complexes showed the vibrational fundamentals of the ligand **BPMT** with some observable shifts which confirm the coordination with the Zn(II) ion. The υ_C=N_ and υ_C=C_ modes were detected at 1593 and 1555 cm^−^^1^, respectively, in the case of the ligand **BPMT**. The υ_C=N_ mode was found to shift to higher wavenumbers of 1626 and 1622 cm^−^^1^ in complexes **1** and **2**, respectively. On the other hand, the υ_C=C_ modes were detected at 1582–1540 cm^−^^1^ and 1555, respectively. A strong intense band was detected at 2082 cm^−^^1^ confirming the presence of the SCN^−^ in the structure of complex **1**. In addition, the molecular and supramolecular structures of **1** are exclusively determined using a single crystal X-ray structure. Additionally, the structure of complex **2** is confirmed using ^1^H NMR spectra where two singlets that appeared at δ 2.37 and 2.69 ppm are related to the two methyls of the pyrazole ring at positions 3 and 5, respectively, and a singlet at δ 4.13 ppm is related to the methoxy group, which are at almost the same position as in the ligand **BPMT**; the CH of pyrazole for complex **2** showed some shift (6.52 ppm) compared to the free ligand (6.05 ppm).

### 2.2. Structure Description of [Zn(BPMT)(NCS)_2_] Complex; (***1***)

**Figure 2:**
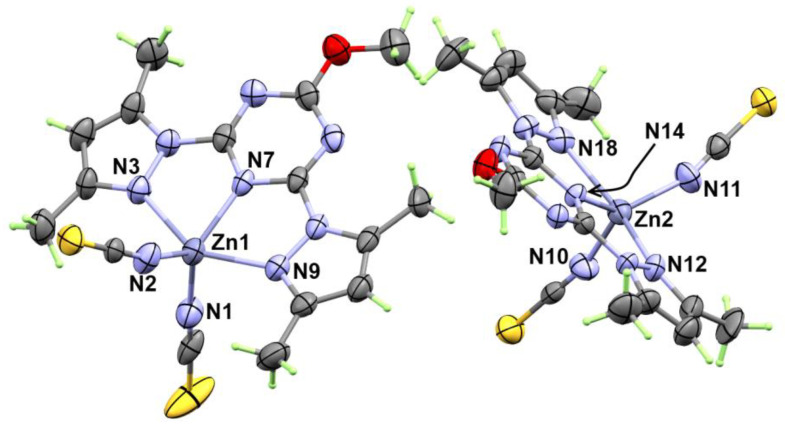
X-ray structure with thermal ellipsoids at 30% probability level for **[Zn(BPMT)(NCS)_2_]**; **1**.

The X-ray structure of **[Zn(BPMT)(NCS)_2_]** (**1**) is shown in Figure 2. This complex crystallized in the monoclinic crystal system with crystal parameters a = 15.518 (5) Å, b = 8.963 (3) Å, c = 16.818 (5) Å and β = 110.531 (11)° (Table S1). The unit cell volume is 2190.6 (12) Å^3^ and Z = 2. The complex crystallized in the *P1c1* space group and two **[Zn(BPMT)(NCS)_2_]** complex units as an asymmetric formula.

The structure of the **[Zn(BPMT)(NCS)_2_]** complex showed a penta-coordinated Zn(II) with one **BPMT** ligand as a tridentate *NNN*-pincer chelate and two terminally coordinated thiocyanate anions (Figure 2). In both molecules found in the asymmetric formula, the bonds Zn1-N7 (2.055 (7) Å) and Zn2-N14 (2.061 (6) Å) with the *s*-triazine core are shorter than the corresponding Zn-N (pyrazole) bonds. In the molecule with lower atom numbering, the Zn1-N9 and Zn1-N3 bonds are found to be 2.191 (7) Å and 2.223 (7) Å, respectively, while in the other unit, the Zn2-N12 and Zn2-N18 are found to be 2.202 (7) and 2.234 (7) Å, respectively. In this complex, the pincer ligand forms two five membered chelate rings with small N-Zn-N bite angles of 72.8 (3)° (N7-Zn1-N9) and 72.9 (3)° (N7-Zn1-N9) in one unit. In the other unit, the bite angles N14-Zn2-N18 and N14-Zn2-N12 are 72.5 (3) and 73.0 (3)°, respectively. The penta-coordination environment of the Zn(II) is completed by two short interactions with two isothiocyanate groups (NSC^−^) via the nitrogen atom while the S-atom sets were freely uncoordinated. Hence, the thiocyanate ion acts as an *N*-terminal ligand. The Zn-N distances with the thiocyanate anions are found to be the shortest among the rest of the Zn-N distances. The Zn1-N1 and Zn1-N2 bond distances are 1.932 (8) Å and 1.938 (9) Å while the corresponding Zn2-N11 and Zn2-N10 bond distances are 1.918 (8) Å and 1.935 (7) Å, respectively. The N1-Zn1-N2 and N10-Zn2-N11 angles are found to be 117.3 (4) and 116.6 (3)°, respectively. In this regard, the *ZnN5* coordination environment [25] is considered highly distorted compared to any of the two ideal geometries of the five coordinated systems; the trigonal bipyramidal and square pyramidal configurations. The τ_5_ parameter ((β − α)/60) was used as a measure for the extent of distortion in the five coordinated metal ion systems. The β and α values are the largest angles in the coordination sphere. Using the results listed in Table 1, the τ_5_ values were calculated to be 0.355 and 0.278 for Zn1 and Zn2, respectively. Hence, the coordination geometry is more likely to be a distorted square pyramidal for both Zn(II) sites.

**Figure 3:**
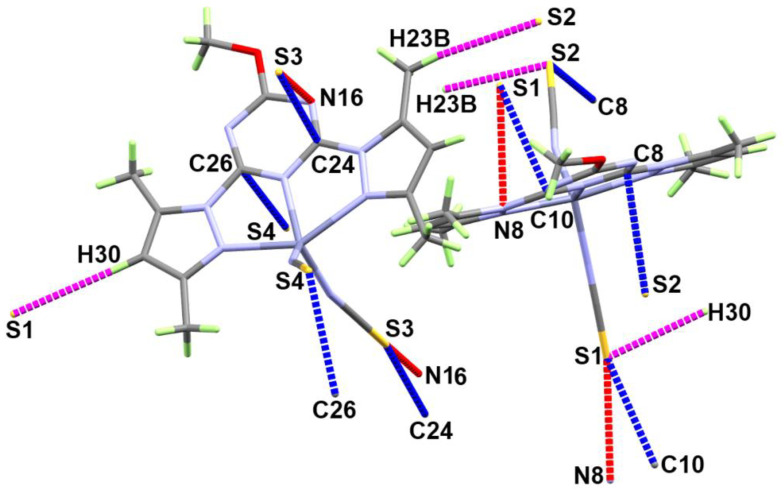
Important contacts in [Zn(BPMT)(NCS)_2_]; **1**.

**Figure 4:**
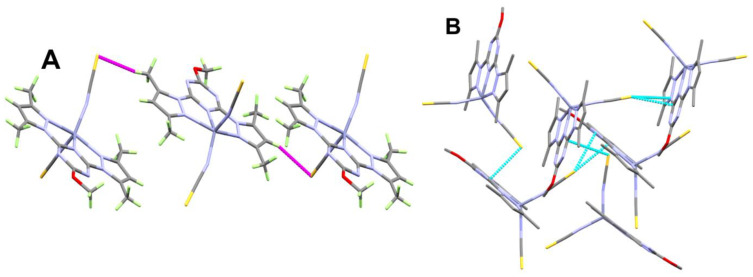
Packing schemes via S…H (**A**) and S…C/S…N (**B**) contacts in **[Zn(BPMT)(NCS)_2_]**; **1**. All hydrogen atoms were omitted from Figure 4B for better clarity.

The supramolecular structure of **[Zn(BPMT)(NCS)_2_]** complex is controlled by the S…H, S…N and S…C contacts shown in Figure 3 while the corresponding contact distances are listed in Table 2. The weak S…H interactions occurred alternatively between the two molecular units found in the asymmetric formula leading to the 1D chain shown in Figure 4A. The hydrogen-acceptor distances of the S2…H23B and S1…H30 interactions are 2.915 and 3.377 Å, respectively. Additionally, there are some intermolecular interactions between the free S-atom of the coordinated thiocyanate ion from one unit with the carbon and nitrogen atoms of the *s*-triazine core from another complex unit. The most important C…S and S…N contacts are given in Table 2. The resulting packing scheme is shown in Figure 4B.

### 2.3. Hirshfeld Analysis

**Figure 5:**
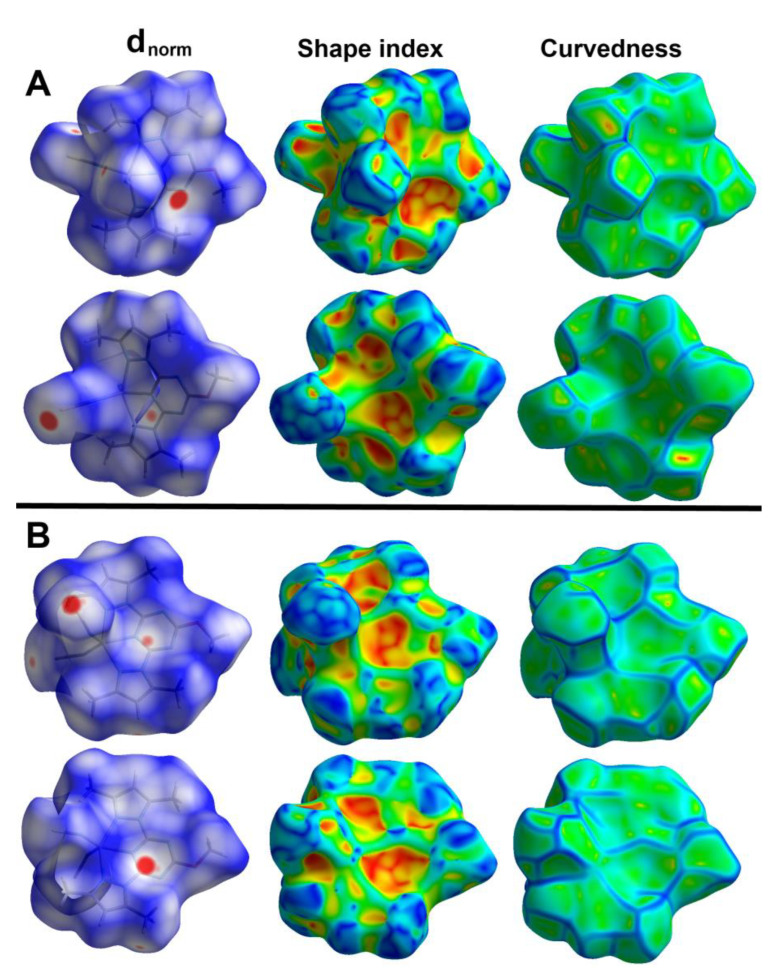
Hirshfeld surfaces of [Zn(BPMT)(NCS)_2_] complex. The Zn1 and Zn2 complex units are designated (**A**,**B**), respectively.

Hirshfeld analysis is an important tool for decomposing all intermolecular interactions in the structure of crystalline materials. For this task, the Crystal Explorer 17.5 program [26] was used to perform the Hirshfeld analysis. The Hirshfeld surfaces of the two complex molecules in the asymmetric unit are shown in Figure 5. Curvedness and shape index maps could give enough evidence of the presence or absence of π-π stacking interactions. The absence of a green flat area in the former and the absence of red/blue triangles in the latter confirm the absence of any π-π stacking interactions in the studied system. 

**Figure 6:**
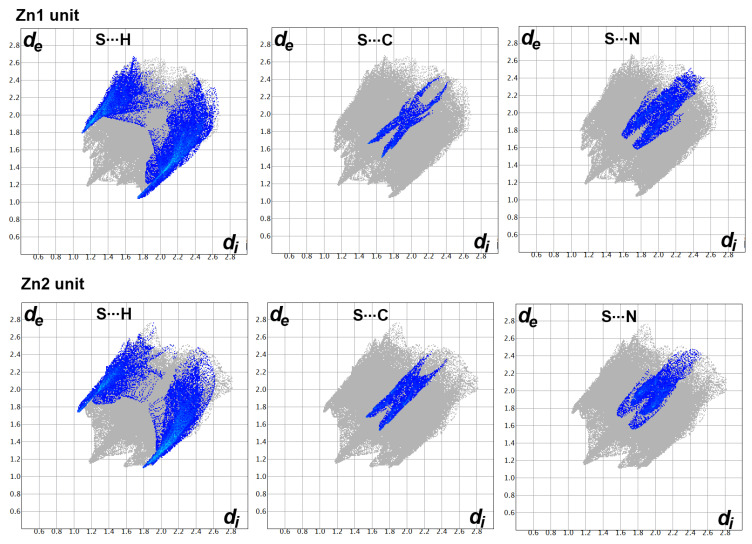
The decomposed fingerprint of the S…H, S…C and S…N interactions in the **[Zn(BPMT)(NCS)_2_]** complex.

On the other hand, there are many red spots in the d_norm_ map corresponding to the S…H, S…C and S…N intermolecular interactions. The percentages of these contacts in the Zn1 unit are 20.2, 3.5 and 5.8%, respectively while the respective values in the Zn2 unit are 18.8, 4.3 and 4.6%. All these intermolecular contacts are significant and appeared as spikes in the fingerprint plots shown in Figure 6.

**Figure 7:**
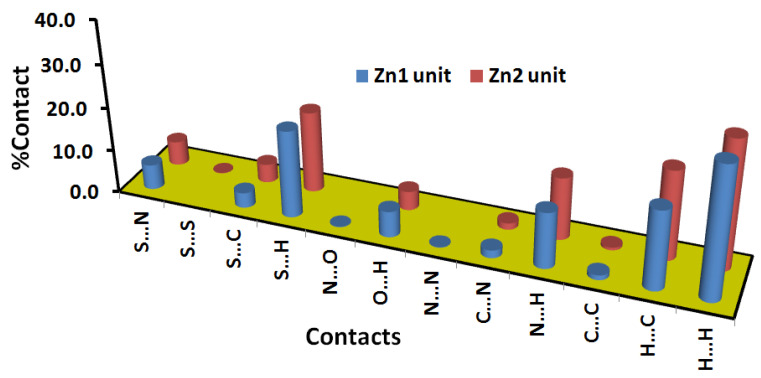
Percentages of all intermolecular contacts in **[Zn(BPMT)(NCS)_2_]** complex.

Additionally, there is large number of weak intermolecular contacts contributing to the molecular packing of this complex (Figure 7). These contacts appeared in the d_norm_ map as blue or white regions. Hence, these contacts have either longer or equal interaction distances than the sum of the vdWs radii of the two interacting atoms. Among these interactions, the H…H, H…C and N…H are the most dominant. Their percentages were found to be 30.3, 18.2 and 12.9% in the Zn1 unit, respectively. In the case of the Zn2 unit, the respective values are 29.7, 20.6 and 14.4%.

### 2.4. Biological Studies

#### 2.4.1. Antimicrobial Activity

The biological activity of the free **BPMT**, complexes **1** and **2** against different microbes including bacteria and fungi were determined by detecting the sizes of inhibition zones at 10 mg/mL of the investigated compounds. All results are listed in Table 3. The free **BPMT** ligand showed only weak antibacterial activity against *S. aureus* with an inhibition zone of 8 mm. On the other hand, complexes **1** and **2** have interesting antimicrobial activity. Both complexes have good antibacterial activity against all the studied microbes to different extents. For complex **1**, the inhibition zone diameters are 16 and 33 mm against *S. aureus* and *B. subtilis*. The corresponding values for complex **2** are 16 and 24 mm. Hence, both complexes have similar antibacterial action against *S. aureus* while complex **1** has better action against *B. subtilis* than complex **2**. For the gram negative bacteria *E. coli* and *P. vulgaris*, complex **1** has better action against both microbes compared to complex **2**. The inhibition zone diameters are 20 and 26 mm for complex **1** against *E. coli* and *P. vulgaris,* respectively. The corresponding values for complex **2** are 13 and 25 mm, respectively. The most promising results are found for complex **1** against *B. subtilis* (33 mm) and *P. vulgaris* (26 mm). For Gentamycin, the respective values for the inhibition zone diameters are 26 and 25 mm. The studied Zn(II) complexes (**1** and **2**) have better antibacterial actions against *S. aureus, B. subtilis* and *E. coli* than the previously reported [Zn(BPMT)(NO_3_)_2_] and [Zn(BPMT)(H_2_O)Cl]ClO_4_ complexes [23]. The inhibition zone diameters for [Zn(BPMT)(NO_3_)_2_] and [Zn(BPMT)(H_2_O)Cl]ClO_4_ are in the range of 11–16 mm against these microbes. In terms of antifungal activity, complex **2** has no activity against the harmful fungi *A. fumigatus* and *C. Albicans* while complex **1** is active only against *C. Albicans* (12 mm). The inhibition zone diameter of Ketoconazole as antifungal control is 20 mm. Hence the studied Zn(II) complexes showed either weak or no antifungal activity against the studied fungi. In comparison with the related Zn(II) complexes [23], the inhibition zone diameters were determined against *C. albicans* to be 16 and 10 mm for [Zn(BPMT)(NO_3_)_2_] and [Zn(BPMT)(H_2_O)Cl]ClO_4_, respectively. Hence, the former has better antifungal action against this microbe than complex **1**. The opposite is true for the perchlorate complex. 

Additionally, the minimum inhibitory concentrations (MIC) in μg/mL were determined. Complex **1** showed interestingly low MIC values against *B. subtilis* (2.4 μg/mL) and *P. vulgaris* (4.8 μg/mL) compared to *Gentamycin* (4.8 μg/mL). Additionally, complex **2** has relatively low MIC values against the same microbes (9.7 and 39.1 μg/mL, respectively (Table 4).

#### 2.4.2. Antioxidant and Anticancer Activities

**Figure 8:**
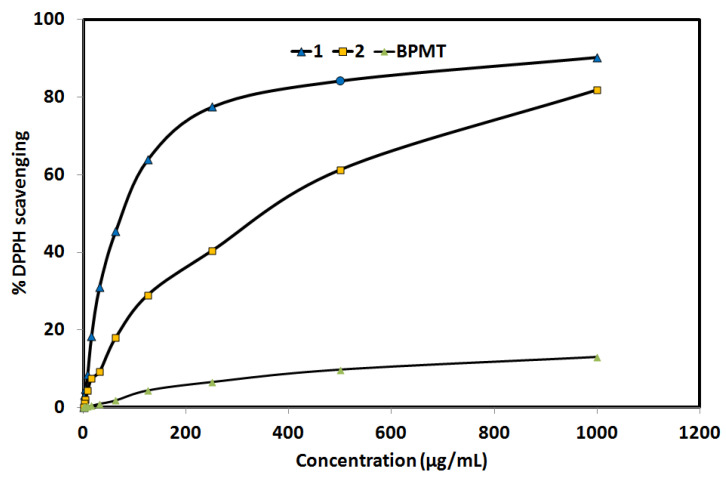
The antioxidant activity of the studied systems. Further details are given in Tables S2–S4.

The DPPH method was used to detect the antioxidant activity of the studied compounds (Figure 8). The free **BPMT** ligand exhibited no antioxidant activity. In contrast, complexes **1** (IC_50_ = 78.09 ± 4.23 µg/mL) and **2** (IC_50_ = 365.60 ± 20.89 µg/mL) showed antioxidant activity which is found to depend on the coordinating anion. It is obvious that the thiocyanate complex (**1**) has better antioxidant activity than the bromo complex (**2**). For the reference ascorbic acid, the IC_50_ value was determined to be 12.3 ± 0.51 µg/mL. Hence, complex **1** has better antioxidant activity than complex **2**. These results indicated that the antioxidant activity of the studied Zn(II) complexes is sensitive to the nature of the coordinating anion.

**Figure 9:**
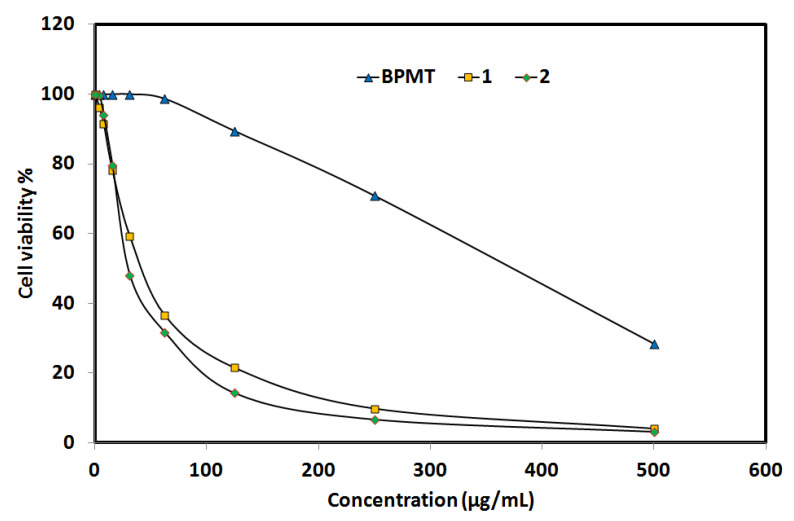
Anticancer activity of the studied systems against lung cancer cell (Details are listed in Tables S5–S7).

In addition, in vitro cytotoxic activity of complexes **1** and **2** against the lung carcinoma A-549 cell line was examined and compared with the free **BPMT** ligand (Figure 9). Both Zn(II) complexes have promising cytotoxic activity against the lung carcinoma A-549 cell line, with enhanced anticancer activity compared to the free **BPMT** ligand. For complexes **1** and **2**, the IC_50_ values are 43.86 ± 3.12 and 30.23 ± 1.26 µg/mL, respectively, while the corresponding value for the free **BPMT** ligand is 372.79 ± 13.64 µg/mL. The studied Zn(II) complexes have 8 and 12 times the anticancer activity of the free **BPMT** ligand, respectively. Hence, complex **2** has the best anticancer activity against the lung carcinoma A-549 cell line. For cis-platin as control, the IC_50_ value is 7.53 ± 0.69 µg/mL indicating moderate anticancer activity for both Zn(II) complexes against the lung carcinoma A-549 cell line.

## 3. Materials and Methods

Chemicals and instrumentations, single crystal X-ray structure measurement details [27,28,29,30,31] and Hirshfeld calculations [26] are presented in Supplementary materials. Synthesis of **BPMT** is described in Method S1 and Scheme S1 (Supplementary Materials). FTIR spectra of **BPMT**, complexes **1** and **2** are given in Figures S1–S3, respectively. NMR spectra of **BPMT** and **2** are shown in Figures S4 and S5, respectively. 

### 3.1. Synthesis of Zn(II) Complexes

The synthesis of the [Zn(BPMT)X_2_] complexes (X = SCN^−^; **1** and X = Br^−^; **2**) was performed by mixing 10 mL ethanolic solution of **BPMT** (29.9 mg, 0.1 mmol) with Zn(NO_3_)_2_·6H_2_O (29.8 mg, 0.1 mmol) in 5 mL distilled water followed by the addition of 1 mL saturated KSCN (for **1**) or KBr (for **2**) aqueous solution. The resulting solutions were left for slow evaporation at room temperature, and colorless crystals of the target compounds were obtained after two weeks. Only for complex **1**, the quality of single crystals was found suitable for the single crystal X-ray structure measurement.

Yield; C_16_H_17_N_9_OS_2_Zn (**1**) 76%. Anal. Calc. C, 39.96; H, 3.56; N, 26.21; S, 13.34; Zn, 13.60%. Found: C, 39.62; H, 3.43; N, 26.01; S, 13.12; Zn, 13.35. IR (KBr, cm^−1^): 3103, 3038, 2990, 2082, 1626, 1539.

Yield; C_14_H_17_N_7_OBr_2_Zn (**2**) 71%. Anal. Calc. C, 32.06; H, 3.27; N, 18.69; Br, 30.47; Zn, 12.47%. Found: C, 31.83; H, 3.19; N, 18.57; Br, 30.32; Zn, 12.31. IR (KBr, cm^−1^): 3098, 2984, 1622, 1582, 1540. ^1^H NMR (DMSO-*d_6_*) δ (ppm): 6.52 (s, 2H, 2*CH-_pyrazole_*), 4.13 (s, 3H, *OCH_3_*), 2.69 (s, 6H, 2*CH_3_*), 2.37 (s, 6H, *2CH_3_*) ppm (Figure S5). 

### 3.2. Biological Studies

The bio-activities of the free **BPMT** and the two Zn(II) complexes as antimicrobial, anticancer and antioxidant agents were investigated. The experimental details are described in Methods S2–S4 (Supplementary Materials).

## 4. Conclusions

Synthesis and biological evaluations of two new penta-coordinated Zn(II) complexes with an *s*-triazine pincer type ligand were presented. Mixing an aqueous solution of Zn(NO_3_)_2_.6H_2_O and ethanolic solution of the **BPMT** pincer ligand in presence of either KSCN or KBr afforded [Zn(BPMT)(NSC)_2_] (**1**) and [Zn(BPMT)(Br)_2_] (**2**) complexes at a good yield. The structure of complex **1** is confirmed by X-ray single crystal structure where the Zn(II) ion is coordinated with one tridentate **BPMT** ligand and two isothiocyanate ions. Its supramolecular structure aspects were analyzed using Hirshfeld analysis. The H…H, H…C and N…H interactions are the most dominant in the crystal structure of **1**. Only the S…H, S…C and S…N contacts have the characteristics of short interactions. The antimicrobial, antioxidant and anticancer activities of the Zn(II) complexes are compared with the results of the **BPMT** ligand. The latter has no or very weak biological activity while the studied Zn(II) complexes are biologically active. It is found that; complex **1** has better antibacterial and antioxidant activities than complex **2**. In contrast, complex **2** (30.23 ± 1.26 µg/mL) has better anticancer activity than complex **1** (43.86 ± 3.12 µg/mL) against the lung carcinoma A-549 cell line.

## Figures and Tables

**Table 1:** Selected bond distances and angles for **1**.

Bond	Distance	Bond	Distance
Zn1-N1	1.932 (8)	Zn2-N11	1.918 (8)
Zn1-N2	1.938 (9)	Zn2-N10	1.935 (7)
Zn1-N7	2.055 (7)	Zn2-N14	2.061 (6)
Zn1-N9	2.191 (7)	Zn2-N12	2.202 (7)
Zn1-N3	2.223 (7)	Zn2-N18	2.234 (7)
**Bond**	**Angle**	**Bond**	**Angle**
**N1-Zn1-N2**	**117.3 (4)**	**N11-Zn2-N10**	**116.6 (3)**
N1-Zn1-N7	118.3 (3)	N10-Zn2-N14	114.7 (3)
N2-Zn1-N7	124.4 (3)	N11-Zn2-N14	128.6 (3)
N1-Zn1-N9	99.7 (3)	N11-Zn2-N12	100.1 (3)
N2-Zn1-N9	100.4 (3)	N10-Zn2-N12	100.8 (3)
N7-Zn1-N9	72.9 (3)	N14-Zn2-N12	73.0 (3)
N1-Zn1-N3	98.1 (3)	N11-Zn2-N18	98.1 (3)
N2-Zn1-N3	97.1 (3)	N10-Zn2-N18	97.1 (3)
N7-Zn1-N3	72.8 (3)	N14-Zn2-N18	72.5 (3)
N9-Zn1-N3	145.7 (3)	N12-Zn2-N18	145.3 (3)

**Table 2:** Intermolecular contacts in **[Zn(BPMT)(NCS)_2_]** complex.

Atoms	Contacts (Å)	Symm. Code
S2…H23B	2.915	x, 1 − y, 1/2 + z
S1…H30	2.939	1 + x, 2 − y, 1/2 + z
N8…S1	3.326	x, −1 + y, z
C10…S1	3.163	x, −1 + y, z
C8…S2	3.377	x, 2 − y, −1/2 + z
N16…S3	3.293	x, −1 + y, z
C24…S3	3.214	x, −1 + y, z
S4…C26	3.393	x, 1 − y, −1/2 + z

**Table 3:** Zone of inhibition (in mm) for **BPMT**, complexes **1** and **2**.

Microbe	1	2	BPMT	Control
*A. fumigatus*	NA ^c^	NA ^c^	NA ^c^	17 ^a^
*C. albicans*	12	NA ^c^	NA ^c^	20 ^a^
*S. aureus*	16	16	8	24 ^b^
*B. subtilis*	33	24	NA ^c^	26 ^b^
*E. coli*	20	13	NA ^c^	30 ^b^
*P. vulgaris*	26	25	NA ^c^	25 ^b^

^a^ Ketoconazole, ^b^ Gentamycin ^c^ Not active.

**Table 4:** MIC values (μg/mL) for complexes **1** and **2**.

Microbe	1	2	Control
*A. fumigatus*	ND ^c^	ND ^c^	156.25 ^a^
*C. albicans*	1250	ND ^c^	312.5 ^a^
*S. aureus*	625	312.5	9.7 ^b^
*B. subtilis*	2.4	9.7	4.8 ^b^
*E. coli*	156.25	1250	4.8 ^b^
*P. vulgaris*	4.8	39.1	4.8 ^b^

^a^ Ketoconazole ^b^ Gentamycin ^c^ Not determined.

## Data Availability

Not applicable.

## Supplementary Materials

The following supporting information can be downloaded at: https://www.mdpi.com/article/10.3390/molecules27113625/s1. Chemicals and instrumentations; X-ray structure measurement details; Figure S1: FTIR spectra of **BPMT;** Figure S2: FTIR spectra of **1**; Figure S3: FTIR spectra of **2;** Figure S4: ^1^H and ^13^C NMR spectra of the ligand (**BPMT**); Figure S5: ^1^H NMR spectra of the complex **2**. Chemical shifts are reported in parts per million (ppm); Scheme S1: Synthesis of the ligand (**BPMT**); Method S1: Synthesis of **BPMT**; Method S2: **Antimicrobial** **studies**; Method S3: **DPPH Radical Scavenging Activity**; Method S4: **Evaluation of Cytotoxic activity**; Table S1: Crystal data and refinement details of **1**; Table S2: Evaluation of antioxidant activity of **1**; Table S3: Evaluation of antioxidant activity of **2;** Table S4: Evaluation of antioxidant activity of **BPMT**; Table S5: Evaluation of cytotoxicity of **1** against A-549 cell line; Table S6: Evaluation of cytotoxicity of **2** against A-549 cell line; Table S7: Evaluation of cytotoxicity of **BPMT** against A-549 cell line [32,33,34].

## Sample Availability

Samples of the compounds are not available from the authors.
