# Supplementary material for: Synthesis, Structure and Biological Evaluations of Zn(II) Pincer Complexes Based on S-Triazine Type Chelator
Source: Molecules. 2022 Jun 5;27(11):3625. doi: 10.3390/molecules27113625 (PMC9182043; doi:10.3390/molecules27113625)
Supplement: Supplementary file 1 [file molecules-27-03625-s001.zip › Supplementary Materials.pdf]

Synthesis, structure and biological evaluations of Zn(II) pincer complexes based on *s*-triazine type chelator

Heba M. Refaat[1]*, Atallh A. M. Alotaibi[1], Necmi Dege[2], Ayman El-Faham[1]*, Saied M. Soliman[1]*

[1]Department of Chemistry, Faculty of Science, Alexandria University, P.O. Box 426, Ibrahimia, Alexandria 21321, Egypt; Emails: hebarefaat212@yahoo.com (H.M.R); Atallh1415@gmail.com (A.A.M.A); aymanel_faham@hotmail.com (A.E-F) and saied1soliman@yahoo.com (S.M.S)

[2]Department of Physics, Faculty of Arts and Sciences, Ondokuz Mayıs University, Samsun, 55139, Turkey; necmid@omu.edu.tr

*Correspondence: hebarefaat212@yahoo.com (H.M.R); aymanel_faham@hotmail.com (A.E-F) and saied1soliman@yahoo.com (S.M.S)

Chemicals and instrumentations

Chemicals were purchased from Sigma-Aldrich Company (Chemie GmbH, 82024 Taufkirchen, Germany). CHNS analyses were performed using Perkin Elmer 2400 Elemental Analyzer (PerkinElmer, Inc.940 Winter Street, Waltham, MA, USA). The bromide content was determined using Volhard's method. The Zn content was determined using Shimadzu atomic absorption spectrophotometer (AA-7000 series, Shimadzu, Ltd, Japan). An Alpha Bruker spectrophotometer (Billerica, Massachusetts, USA) was used to measure the FTIR spectra in KBr pellets (**Figs. S1-S3**). Nuclear magnetic resonance spectra measured on 400 MHz JEOL (JEOL, Ltd., Tokyo, Japan) spectrometer (**Figs. S4** and **S5**).

X-ray structure measurement details

For X-Ray diffraction analysis, suitable crystals were selected and data collection was performed on a Bruker diffractometer equipped with a graphite-monochromatic Mo-Kα radiation at 296 K. The structures were solved by direct methods using SHELXT-2018 [27] and refined by full-matrix least-squares methods on F^2 using SHELXL-2018 [28] from within the WINGX [29] suite of software. Bruker APEX2 [30] was used for data collection, while molecular diagrams were created using MERCURY [31]. Hirshfeld calculations were performed using Crystal Explorer 17.5 program [26].

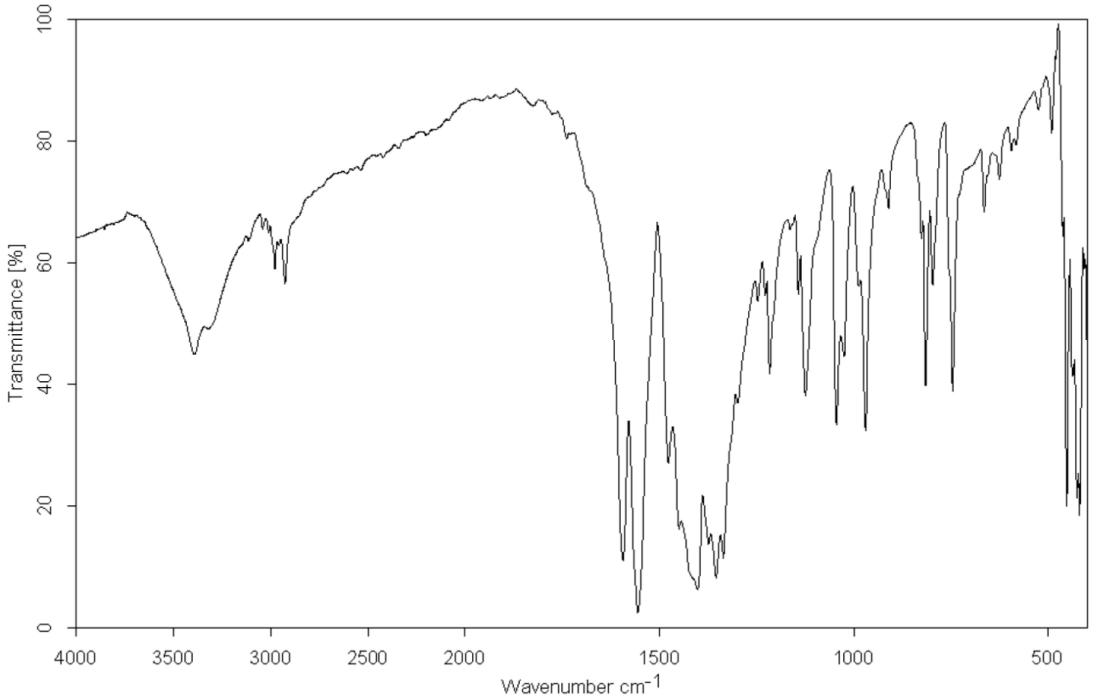

Figure S1. FTIR spectra of **BPMT**.

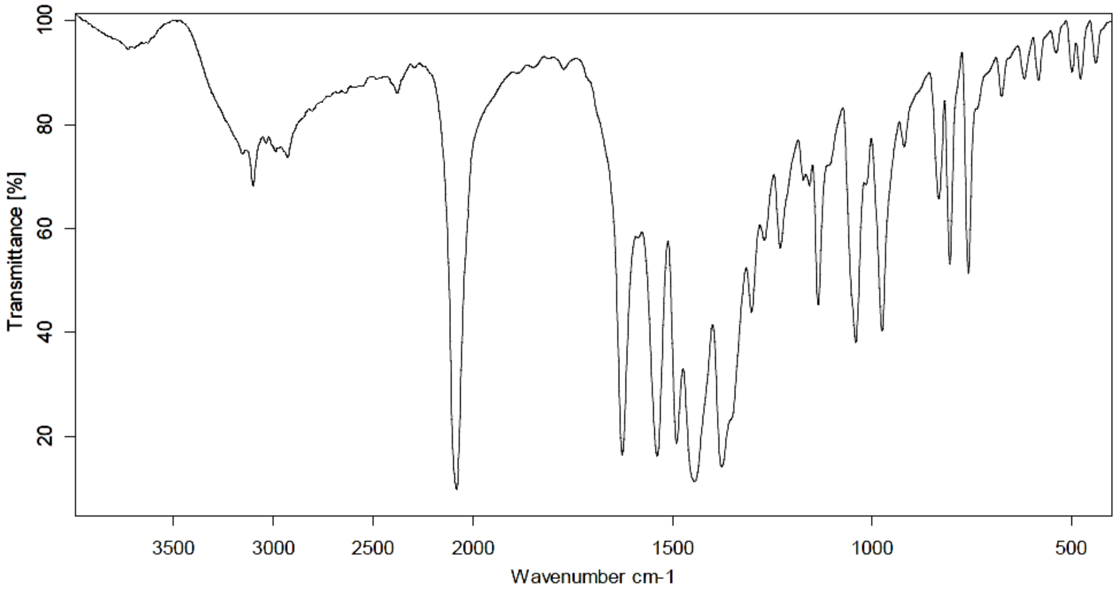

Figure S2. FTIR spectra of **1**.

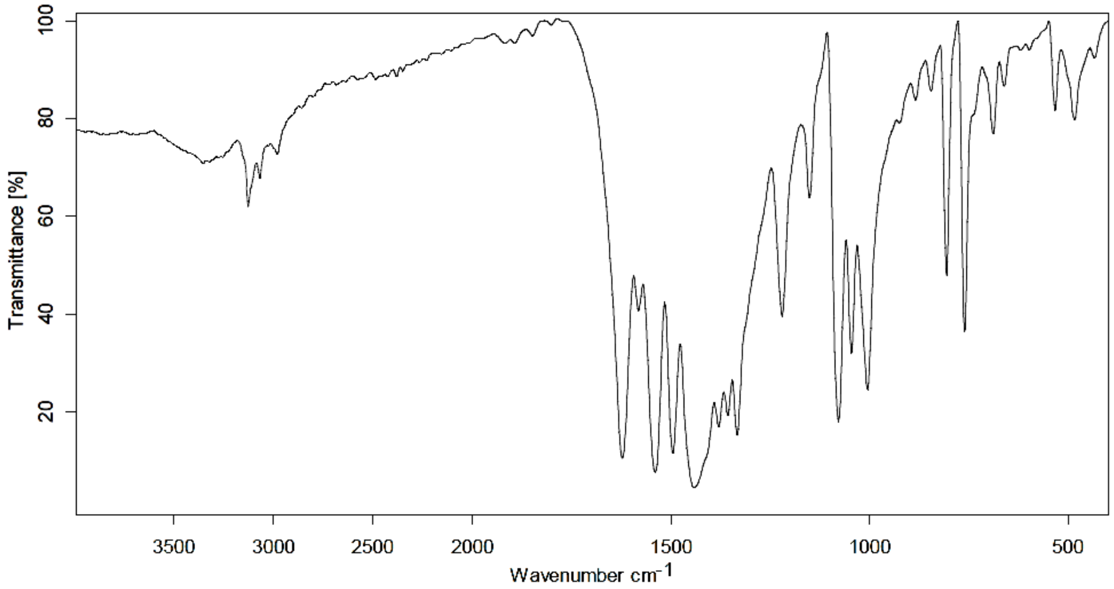

Figure S3. FTIR spectra of **2**.

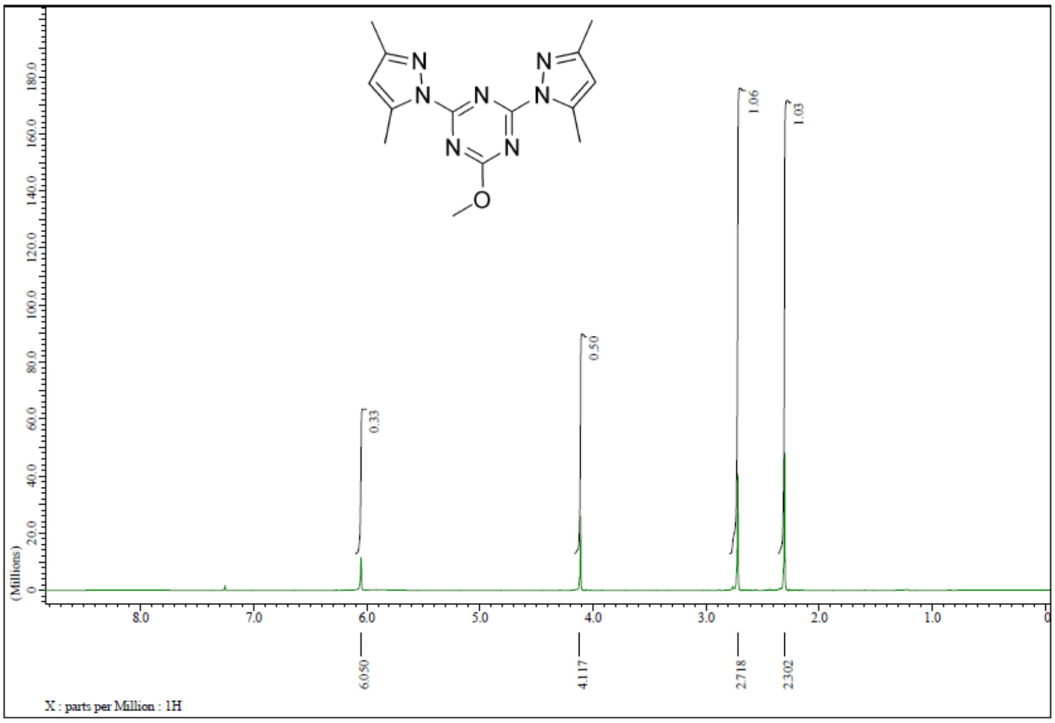

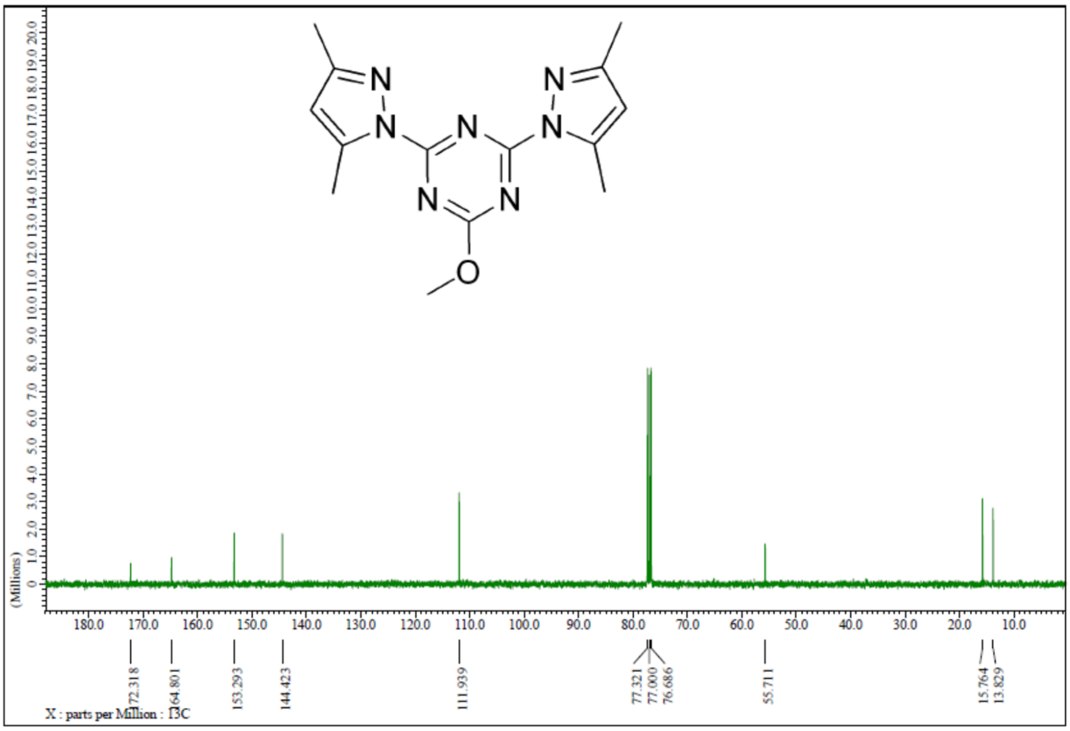

Figure S4. ^{1}H and ^{13}C NMR spectra of the ligand (**BPMT**). Chemical shifts are reported in parts per million (ppm).

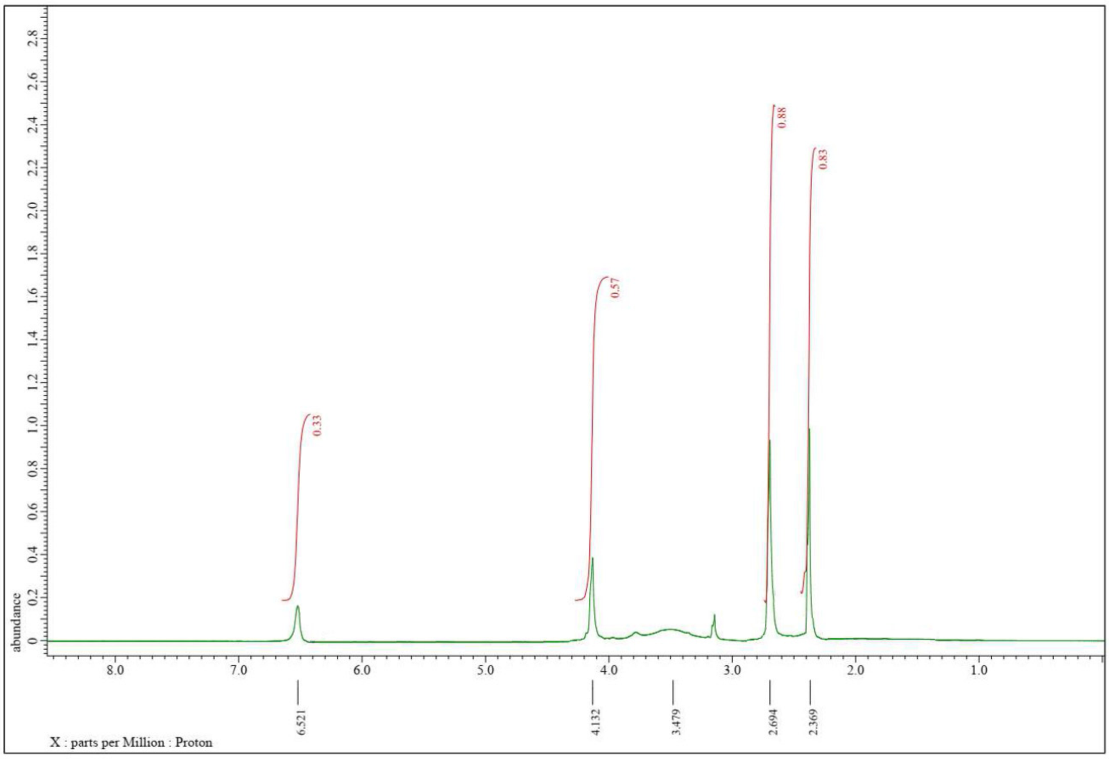

Figure S5. ^{1}H NMR spectra of the complex **2**. Chemical shifts are reported in parts per million (ppm).

Method S1: Synthesis of BPMT [11, 12]

The ligand (**BPMT**) was synthesized following the method shown in **Scheme S1**. At room temperature, 2,4-dihydrazinyl-6-methoxy-1,3,5-triazine (10 mmol in 20 mL DMF) was mixed with acetylacetone (25 mmol) followed by addition of triethylamine (8 mmol in 10 mL DMF) with stirring then refluxed for 6 h. After completion of the reaction, the mixture is left to cool till room temperature, and then ice-cold water was added (100 mL). The reaction mixture was kept at 0°C for 2 h, white solid powder of **BPMT** is precipitated. The product is filtered, washed with cold water (3 x 20 mL), and then dried.

Yield; $C_{14}N_7H_{17}O$ (**BPMT**) 86%; mp 175-176 °C. IR (KBr, cm^{-1}): 3041, 2978, 2926, 1593, 1555. ^{1}H NMR ($CDCl_3$) δ: 2.30 (s, 6H, $2CH_3$), 2.72 (s, 6H, $2CH_3$), 4.12 (s, 3H, OCH_3),6.05 (s, 2H, CH) ppm; ^{13}C NMR ($CDCl_3$): δ 13.8, 17.8, 55.7, 111.9, 144.4, 153.3, 164.8, 172.3 ppm.

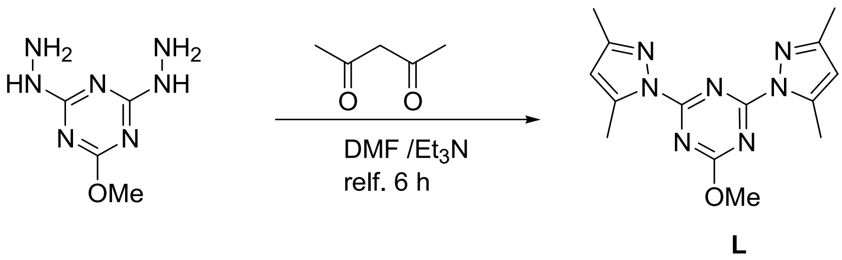

Scheme S1. Synthesis of the ligand (**BPMT**).

Method S2: Antimicrobial studies [S1]

a) Tested pathogenic microbes

The antibacterial activity of the studied ligand and their Zn(II) complexes were evaluated against two Gram positive bacteria (*S. aureus* (ATCC 25923)) and *B. subtilis* (RCMB015(1)NRR LB-543)), two Gram negatvie bacteria (*E. coli* (ATCC 25922)) and *P. vulgaris* (RCMB 004(1)ATCC 13315)) and two fungi (*A. fumigatus* (RCMB 002008)) and *C. albicans* (RCMB 005003(1) ATCC 10231)). Gentamycin was used as standard antibacterial agent. The samples maintained in Brain heart infusion (BHI) at 20°C; 300 mL of each stock–culture was added to 3 mL of BHI broth. Overnight cultures were kept for 24 h at 37 °C ± 1°C and the purity of cultures was checked after 24 h of incubation. After 24 h of incubation, bacterial suspension was diluted with sterile physiological solution, for the diffusion and indirect bioautographic tests, to 108 CFU/mL (turbidity = McFarland barium sulfate standard 0.5). In case of fungi *A. fumigatus* (RCMB 002008) and *C. albicans* (RCMB 005003(1) ATCC 10231), the used medium in antagonistic activity against tested fungi is Potato Dextrose Agar, where Fluconazole was used standard antifungal agent.

b) Agar well diffusion method

Synthetic compounds were prepared at concentration 10 mg/mL dissolved in DMSO as stock solutions. Preparation of sterilized Mueller Hinton agar plates seeded with tested pathogenic bacteria occurred. The wells are done by sterilized cork borer in size 6 mm and hence 200 μg of the synthetic compound was poured in each well comparably with DMSO as control. The plates were incubated at 37°C for 24 h. after incubation period; antimicrobial activity was determined by inhibition zones.

c) <u>Minimum Inhibitory Concentration (MIC)</u>

Different dilutions of the compounds are inoculated with tested pathogenic microbes. After incubation period of 96 well microplate, the results are measured using microplate reader. To determine at what level the MIC endpoint is established; subculture of test samples at different concentrations occurred in nutrient agar plates.

<u>Method S3 DPPH Radical Scavenging Activity [S2]</u>

Freshly prepared (0.004%w/v) methanol solution of 2,2-diphenyl-1-picrylhydrazyl (DPPH) radical was prepared and stored at 10 ºC in dark place. A methanolic solution of the test compound was prepared using the same procedure. A 40 uL aliquot of the methanol solution was added to 3mL of DPPH solution. Absorbance measurements were recorded immediately with a UV-visible spectrophotometer (Milton Roy, Spectronic 1201). The decrease in absorbance at 515 nm was determined continuously, with data being recorded at 1 min intervals until the absorbance stabilized (16 min). The absorbance of the DPPH radical without antioxidant (control) and the reference compound ascorbic acid were also measured. All the determinations were performed in three replicates and averaged. The percentage inhibition (PI) of the DPPH radical was calculated according to the formula:

$$PI = [\{(AC - AT)/ AC\} \times 100] \quad (1)$$

Where AC = Absorbance of the control at t = 0 min and AT = absorbance of the sample+DPPH at t = 16 min. The 50% inhibitory concentration (IC_{50}), the concentration required to inhibit DPPH radical by 50%, was estimated from graphic plots of the dose response curve.

Method S4 Evaluation of Cytotoxic activity[S3]

Cell line Propagation

The cells were propagated in Dulbecco's modified Eagle's medium (DMEM) supplemented with 10% heat-inactivated fetal bovine serum, 1% L-glutamine, HEPES buffer and 50μg/mL Gentamycin. All cells were maintained at 37ºC in a humidified atmosphere with 5% CO_2 and were subcultured two times a week.

Cytotoxicity evaluation using viability assay

For cytotoxicity assay, the cells were seeded in 96-well plate at a cell concentration of 1×10^4 cells per well in 100μl of growth medium. Fresh medium containing different concentrations of the test sample was added after 24 h of seeding. Serial two-fold dilutions of the tested chemical compound were added to confluent cell monolayers dispensed into 96-well, flat-bottomed microtiter plates (Falcon, NJ, USA) using a multichannel pipette. The microtiter plates were incubated at 37ºC in a humidified incubator with 5% CO_2 for a period of 24 h. Three wells were used for each concentration of the test sample. Control cells were incubated without test sample and with or without DMSO. The little percentage of DMSO present in the wells (maximal 0.1%) was found not to affect the experiment. After incubation of the cells for at 37°C, for 24 h, the viable cells yield was determined by the MTT test. Briefly, the media was removed from the 96 well plate and replaced with 100 μL of fresh culture DMEM medium without phenol red then 10 μL of the 12 mM MTT stock solution (5 mg of MTT in 1 mL of PBS) to each well including the untreated controls. The 96 well plates were then incubated at 37°C and 5% CO_2 for 4 hours. An 85 μL aliquot of the media was removed from the wells, and 50 μL of DMSO was added to each well and mixed thoroughly with the pipette and incubated at 37°C for 10 min. Then, the optical density was measured at 590 nm with the microplate reader (SunRise, TECAN, Inc, USA) to determine the number of viable cells and the percentage of viability was calculated as [(ODt/ODc)]x100%

where ODt is the mean optical density of wells treated with the tested sample and ODc is the mean optical density of untreated cells. The relation between surviving cells and drug concentration is plotted to get the survival curve of each tumor cell line after treatment with the specified compound. The 50% inhibitory concentration (IC_{50}), the concentration required to cause toxic effects in 50% of intact cells, was estimated from graphic plots of the dose response curve for each conc. using Graphpad Prism software (San Diego, CA. USA).

References

[S1] CLSI; Clinical and Laboratory Standards Institute, 2012, Twentieth informational supplement. M100-S22. Wayne: PA.

[S2] Yen, G.C. and Duh, P.D. (1994). Scavenging effect of methanolic extracts of peanut hulls on free radical and active oxygen species, *J Agric Food Chem*, 42: 629-632.

[S3] Mosmann, T. (1983): Rapid colorimetric assay for cellular growth and survival: application to proliferation and cytotoxicity assays. J. Immunol. Methods; 65: 55-63.

Table S1. Crystal data and refinement details of **1**.

**CCDC**	**2153437**	
Empirical formula	$C_{16}H_{17}N_9OS_2Zn$	
Formula weight	480.87 g/mol	
Temperature	293(2) K	
Wavelength	0.71073 Å	
Crystal system	Monoclinic	
Space group	P1c1	
Unit cell dimensions	a = 15.518(5) Å	α = 90°
	b = 8.963(3) Å	β = 110.531(11)°
	c = 16.818(5) Å	γ = 90°
Volume	2190.6(12) $Å^3$	
Z	4	
Density (calculated)	1.458 g/cm^3	
Absorption coefficient	1.338 mm^{-1}	
F(000)	984	
Theta range for data collection	2.27 to 28.50°	
Index ranges	$-20 \leq h \leq 20$, $-11 \leq k \leq 11$, $-22 \leq l \leq 22$	
Reflections collected	40799	
Independent reflections	10718 [R(int) = 0.0728]	
Completeness to theta = 28.50°	98.80%	
Refinement method	Full-matrix least-squares on F^2	
Data / restraints / parameters	10718 / 2 / 534	
Goodness-of-fit on F^2	0.997	
Final R indices [I>2sigma(I)]	R1 = 0.0554, wR2 = 0.0820	
R indices (all data)	R1 = 0.1625, wR2 = 0.1069	
Largest diff. peak and hole	0.520 and -0.456	

Table S2. Evaluation of antioxidant activity of **1**.

Sample conc. (µg/mL)	DPPH scavenging %	S.D.
1000	90.34	1.62
500	84.26	1.48
250	77.51	2.13
125	63.90	2.42
62.5	45.38	2.78
31.25	31.12	1.46
15.6	18.46	0.32
7.8	8.39	0.45
3.9	4.75	0.71
2	3.17	0.29
0	0	

Table S3. Evaluation of antioxidant activity of **2**.

Sample conc. (µg/mL)	DPPH scavenging %	S.D.
1000	81.79	2.03
500	61.23	2.91
250	40.34	3.12
125	28.95	1.79
62.5	17.92	0.84
31.25	9.21	0.63
15.6	7.46	0.72
7.8	4.29	0.37
3.9	2.08	0.46
2	0.97	0.31
0	0	

Table S4. Evaluation of antioxidant activity of **BPMT**.

Sample conc. (µg/mL)	DPPH scavenging %	S.D.
1000	12.94	0.88
500	9.71	0.73
250	6.52	0.46
125	4.36	0.32
62.5	1.79	0.17
31.25	0.92	0.24
15.6	0.41	0.23
7.8	0.19	0.07
3.9	0.07	0.04
2	0.03	0.02
0	0	

Table S5. Evaluation of cytotoxicity of **1** against A-549 cell line.

Sample conc. (µg/mL)	Viability %	Inhibitory %	S.D. (±)
500	4.03	95.97	0.59
250	9.78	90.22	1.26
125	21.49	78.51	1.37
62.5	36.52	63.48	2.46
31.25	59.13	40.87	2.91
15.6	78.04	21.96	1.68
7.8	91.37	8.63	1.01
3.9	96.25	3.75	0.69
0	100	0	

Table S6. Evaluation of cytotoxicity of **2** against A-549 cell line.

Sample conc. (µg/mL)	Viability %	Inhibitory %	S.D. (±)
500	3.19	96.81	0.65
250	6.73	93.27	0.31
125	14.28	85.72	0.64
62.5	31.74	68.26	1.98
31.25	48.06	51.94	2.42
15.6	79.53	20.47	2.39
7.8	94.12	5.88	0.74
3.9	99.57	0.43	0.19
0	100	0	

Table S7. Evaluation of cytotoxicity of **BPMT** against A-549 cell line.

**Sample conc. (µg/mL)**	**Viability %**	**Inhibitory %**	**S.D. (±)**
500	28.39	71.61	1.97
250	70.86	29.14	3.12
125	89.43	10.57	2.09
62.5	98.70	1.3	0.68
31.25	100	0	
15.6	100	0	
7.8	100	0	
3.9	100	0	
0	100	0	
